# Efficacy of Tigecycline as Salvage Therapy in Multidrug-Resistant Febrile Neutropenia in Patients with Acute Leukemia—A Single Center Analysis

**DOI:** 10.3390/antibiotics11020128

**Published:** 2022-01-19

**Authors:** Franziska Modemann, Steffen Härterich, Julian Schulze zur Wiesch, Holger Rohde, Nick Benjamin Lindeman, Carsten Bokemeyer, Walter Fiedler, Susanne Ghandili

**Affiliations:** 1 Department of Oncology, Hematology and Bone Marrow Transplantation with Section Pneumology, University Cancer Center Hamburg, University Medical Center Hamburg-Eppendorf, Martinistraße 52, 20246 Hamburg, Germany; nickben97@aol.de (N.B.L.); c.bokemeyer@uke.de (C.B.); fiedler@uke.de (W.F.); s.ghandili@uke.de (S.G.); 2 Mildred Scheel Cancer Career Center, University Cancer Center Hamburg, University Medical Center Hamburg-Eppendorf, Martinistraße 52, 20246 Hamburg, Germany; 3 Hospital Pharmacy, University Medical Center Hamburg-Eppendorf, Martinistraße 52, 20246 Hamburg, Germany; s.haerterich@uke.de; 4 Department of Internal Medicine, Division of Infectious Diseases, University Medical Center Hamburg-Eppendorf, Martinistraße 52, 20246 Hamburg, Germany; j.schulze-zur-wiesch@uke.de; 5 The Institute of Medical Microbiology, Virology and Hygiene, University Medical Center Hamburg-Eppendorf, Martinistraße 52, 20246 Hamburg, Germany; rohde@uke.de

**Keywords:** febrile neutropenia, tigecycline, acute leukemia, multidrug resistant fever

## Abstract

Severe infectious complications remain the main cause of mortality in leukemia patients due to a long period of profound neutropenia. Standardized regimens for antimicrobial, antifungal, and antiviral prophylaxis and therapy in neutropenic patients have improved infection-associated mortality. Nevertheless, many patients are refractory to these multidrug approaches. Tigecycline is a last-resort antibiotic with a broad-spectrum activity; unfortunately, clinical experience in multidrug-resistant febrile neutropenia is limited. The aim was to evaluate the efficacy of tigecycline treatment in comparison to standard treatment in this patient cohort. In this single center analysis, we analyzed the clinical courses of 73 patients with acute leukemia and diagnosis of febrile neutropenia resistant to hospital-based multidrug escalation levels who continued on a standard approach without antibiotics as the last resort (*n* = 30) or were switched to tigecycline in addition to carbapenem treatment (*n* = 43). We observed comparable overall response rates (decrease in C-reactive protein or resolution of fever) in both patient cohorts. Switching the antibiotic approach to tigecycline showed lower absolute sepsis (33% vs. 47%, *p* = 0.235) and infection-associated mortality rates (5% vs. 13%, *p* = 0.221). Prospective larger randomized studies are necessary to underline these results and to be able to generate reliable statistics.

## 1. Introduction

Severe infections and neutropenic fever of unknown origin (FUO) are among the most common complications in patients with acute leukemia (AL) especially in those patients who are undergoing intensive chemotherapy [1,2]. With an FUO associated in-hospital mortality of 14.3%, leukemia patients display the highest febrile neutropenia-related death rates among all cancer types [3]. Especially, a duration of profound neutropenia—defined as <100 neutrophiles/µL [4]—of more than seven days duration is a risk factor for severe bacterial, viral, or fungal infections [5]. The main sites of infection in neutropenic patients are pneumonia and mucositis of the entire gastrointestinal tract [2,6].

According to the latest update of the ASCO (American Society of Clinical Oncology) and IDSA (Infectious Diseases Society of America) guidelines, it is recommended to administer a prophylactic antimicrobial agent in this patient cohort [4]. Since the clinical detection of an infection focus or the detection of pathogenic microbes is mostly unsuccessful in patients with prolonged and profound neutropenia [7,8] and since *Pseudomonas* spp. related infections are associated with high mortality rates [9], effective empirical antibiotic therapies against these bacteria remain the agents of choice for prophylactic treatment and are the backbone for the therapy of neutropenic fever in these patients [4,10,11]. The recommendation for immediate antibacterial therapy within <1 h after onset of FUO reflects the importance of immediate treatment of FUO in patients with AL during prolonged and profound neutropenia [10]. For prophylactic treatment, quinolone therapy remains the standard approach mainly due to its oral administration route and effectiveness against *Pseudomonas* spp. [12]. For deteriorating patients with febrile neutropenia and unknown causative agent, an empiric switch to third generation cephalosporines or piperacillin/tazobactam as the first step, and carbapenem-based therapies as the second step are recommended [13]. Additionally, gram-positive organisms are covered by the addition of vancomycin or linezolid because gram-positive organisms account for the majority (75–80%) of bloodstream infections in patients with neutropenic fever [2,13,14]. These multidrug approaches cover most microorganisms, nevertheless, there are some patients who do not respond to treatment and show no resolution of fever, an increase of inflammation parameters, or a clinical deterioration. This is the patient cohort with multidrug-resistant febrile neutropenia to be investigated in this study. In the era of increasing resistance against several antibiotic drugs, last-resort antibiotics like daptomycin, gentamicin, or tigecycline gain more attention in treating highly affected patients with neutropenic fever or for target-oriented therapeutic approaches. Tigecycline is an antibiotic agent of the glycylcycline group with broad-spectrum activity against gram-positive bacteria, notably also against methicillin-resistant *Staphylococcus aureus* (MRSA) and vancomycin-resistant *Enterococcus* spp. (VRE). Moreover, tigecycline is also effective against gram-negative bacteria, including *Stenotrophomonas maltophilia* and multiresistant *Acinetobacter baumannii* [15]. However, tigecycline is not effective against any *Pseudomonas* spp. [15]; therefore, in high-risk patients, tigecycline must be combined with *Pseudomonas* spp.-active antibiotics. Since neutropenic fever can be a potentially life-threatening condition, the use of reserve antibiotics for multidrug-resistant fever is not unusual in hemato-oncological clinical practice. There are very few studies available which investigated the efficacy of tigecycline in patients with acute leukemia and neutropenic fever. Beside the broad spectrum of activity mentioned above, tigecycline in general is well tolerable and does not require dose adjustment in critically ill patients with poor kidney function or mild to moderate liver problems [16]. Therefore, the efficacy of tigecycline in patients with acute leukemia and multidrug-resistant febrile neutropenia is investigated in this study.

In 2014, Bucaneve et al. demonstrated the superiority of piperacillin/tazobactam combined with tigecycline as a first-line empiric antibiotic therapy compared to piperacillin/tazobactam alone in patients with hematological malignancies and high-risk neutropenia [17]. Results of multiple randomized phase III trials have demonstrated that tigecycline-monotherapy is at least as effective as (a) vancomycin and aztreonam therapy for complicated skin and skin structure infections, (b) imipenem for complicated intraabdominal infections, and (c) levofloxacin for community-acquired bacterial pneumonia. Therefore, tigecycline has been approved, since 2005, for the treatment of complicated skin and skin structure infections and complicated intraabdominal infections as first-in-class glycylcycline, and since 2018, for community-acquired bacterial pneumonia [18,19,20,21]. Because of low serum levels, the use of tigecycline in patients with bacteriemia is controversially discussed [22]. Nevertheless, the use of pseudomonas-active empirical antibiotics in combination with tigecycline in hematological patients with severe neutropenia and with symptoms of severe infection is not part of the clinical routine, since the application of vancomycin or linezolid is preferred due to missing data on the use of tigecycline and also due to the generally restrained application of tigecycline as a last-resort antibiotic.

This retrospective study aimed to investigate the efficacy of tigecycline in combination with pseudomal effective empirical antibiotics as salvage therapy in multidrug-resistant febrile neutropenia compared to the standard of care in patients with acute leukemia and high-risk neutropenia.

## 2. Results

The screened overall study population was divided into two groups: the control group and the tigecycline group. In total, 1415 cases of AML and ALL patients were screened for the study. In 179 patient cases, tigecycline therapy was administered during the analyzed period but only 43 cases met inclusion criteria for the study (refer to materials and methods). For the control group, 30 cases could be included after the initial screening of 1236 AML and ALL patient cases. A total of 58% (*n* = 25) in the tigecycline group were male and the median age was 59 years (IQR 43–68). A total of 86% (*n* = 37) of the patients in the tigecycline group had an AML as underlying disease and 88% (*n* = 38) were treated with intensive chemotherapy. A total of 77% (*n* = 33) had a newly diagnosed leukemia (for patient characteristics, refer to Table 1).

Baseline characteristics of patients in the control group were comparable: 53% (*n* = 16) were male and the median age was 60.5 years (IQR 44–68). A total of 83% patients (*n* = 25) had an AML and 93% of them (*n* = 28) were treated with intensive chemotherapy. A total of 83% of the patients (*n* = 25) had newly diagnosed leukemia.

The Charlson-Comorbidity-Index (CCI) [23] was used to score the comorbidities in both patient cohorts. Each case was scored with an additional 2 points due to underlying leukemia. The two groups also differed little in terms of comorbidities: median CCI in the tigecycline group was 4 (IQR 2–5), and in 11 cases (26%) relevant comorbidities were identified. Median CCI in the control group was 4 (IQR 2–5) as well, and relevant comorbidities were seen in 7 cases (23%) (for detailed information refer to Table 1). The duration of profound neutropenia was comparable between the 2 treatment groups with 25 days in the control group (IQR 20–28 days) and 21 days (IQR 17–30 days) in the tigecycline group.

As defined in the inclusion criteria, each patient was treated with a carbapenem and vancomycin or linezolid at baseline. In the tigecycline group, meropenem was administered in all cases (*n* = 43, 100%) and vancomycin in 41 (95%) of cases, whereas 13 patients (30%) were treated with linezolid. It should be noted that 11 patients (26%) received both vancomycin and linezolid; that is, their treatment started with vancomycin but was switched to linezolid mainly due to nephrotoxicity or allergic reaction. For antifungal therapy in the tigecycline group, 36 cases (84%) were treated with amphotericin B, 5 cases (12%) with caspofungin, and 2 cases (5%) with voriconazole. In the control group, 3 cases (10%) received imipenem/cilastatin instead of meropenem; vancomycin was administered in 26 cases (87%) and linezolid in 13 cases (43%). A total of 10 cases (33%) were treated with both agents. For antifungal treatment (in the control group), 28 cases were treated with amphotericin B, and only for 1 case (3%) was caspofungin or voriconazole administered, respectively. Each patient in both treatment groups received prophylactic acyclovir treatment (for overview of antibiotic treatment distributions, refer to Table 2).

**Figure 1:**
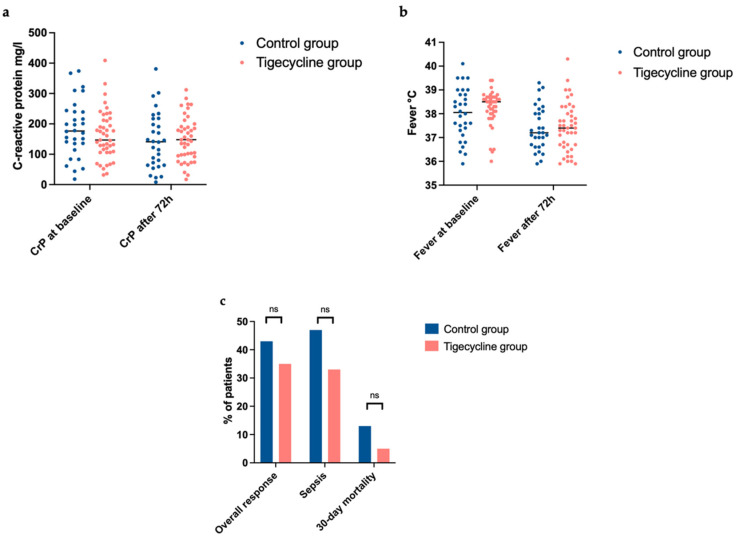
(**a**–**c**) CrP-levels at baseline and after 72 h of treatment in the control group (blue dots) and in the tigecycline group (red dots). Median CrP-level at baseline in the control group was 177 mg/L (IQR 130–241 mg/L) compared to 141 mg/L (IQR 64–217 mg/L) after 72 h. Median CrP-level at baseline in the tigecycline group was 147 mg/L (IQR 106–212 mg/L) compared to 148 mg/L (IQR 95–191 mg/L) after 72 h treatment. (**b**) Fever temperature in °C at baseline and after 72 h of treatment in the control group (blue dots) and in the tigecycline group (red dots). In the control group, median fever temperature was 38.1 °C (IQR 37.3–38.9 °C) at baseline compared to 37.2 °C (IQR 36.7–38.1 °C) after 72 h. In the tigecycline group, median fever temperature was 38.5 °C (IQR 38.0–38.7 °C) at baseline and 37.4 °C (IQR 36.7–38.1 °C) after 72 h. (**c**) Primary and secondary endpoints: Overall response rate (resolution of fever < 38 °C or decrease of CrP level > 10% compared to baseline after 72 h of treatment) was comparable between the control group (43%) and the tigecycline group (35%). A higher rate of sepsis and infection-associated mortality could be observed in the control group compared to the tigecycline group (47% vs. 33%, 13% vs. 5%, not significant, respectively). Patient numbers are indicated in % of all patients in each group. CrP = C-reactive protein; ns = not significant.

The statistical analysis of the primary endpoint of the overall response rate did not show significant differences between the two groups (*p* = 0.476; for characteristics of disease courses and detected microbes, refer to Table 3); an overall response could be observed in 15 cases (35%) of the tigecycline group, and an overall response was observed compared to 13 cases (43%) in the control group. Neither the rate of resolution of neutropenic fever nor of decreased CrP levels were significantly higher in one of the groups: Median CrP-level at baseline in the control group was 177 mg/L (IQR 130–241 mg/l) compared to median CrP level of 141 mg/L (IQR 64–217 mg/l) after 72 h. For the tigecycline group, median CrP-level at baseline was 147 mg/L (IQR 106–212 mg/L) and after 72 h of treatment, median CrP-level was 148 mg/L (IQR 95–191 mg/l). In the control group, the median fever temperature was 38.1 °C (IQR 37.3–38.9 °C) at baseline compared to a median of 37.2 °C (IQR 36.7–38.1 °C) after 72 h. The same slight decrease could be observed in the tigecycline group with a median fever temperature of 38.5 °C (IQR 38.0–38.7 °C) at baseline and 37.4 °C (IQR 36.7–38.1 °C) after 72 h of treatment (for visualization of these results, refer to Figure 1a,b). The rate of sepsis according to the Third International Consensus Definition [24] was higher in the control group (47% (*n* = 14) vs. 33% (*n* = 14), *p* = 0.235, not significant) while more cases of pneumonia occurred in the tigecycline group (60% vs. 53%). Infection-associated 30-day mortality starting from the baseline timepoint was higher in the control group (13% (*n* = 4) vs. 5% (*n* = 2), *p* = 0.221, not significant; for visualization of results, refer to Figure 1c). As usual in the standard clinical routine of patients with neutropenic fever, blood culture samples were collected in every patient during the analyzed period. In one case, a vancomycin-resistant *Enterococcus faecium* could be detected in the tigecycline group and in another case in the control group, a tigecycline-resistant *Staphylococcus haemolyticus* was detected (for infection-associated complications refer to Table 3). In both cases, no treatment adjustment was necessary and both bacteria were found after the treatment decision.

## 3. Discussion

The therapy of neutropenic fever with unknown origin in leukemia patients with long-lasting profound neutropenia is still challenging, although there are standardized approaches and escalation schedules for this patient cohort. We hypothesized that the application of tigecycline in patients with acute leukemia with multidrug-resistant neutropenic fever would lead to a better response rate to antibacterial treatment than the standard regimens with antifungal treatment, antiviral prophylaxis, and carbapenem plus vancomycin or linezolid. There are very limited data available to address this question: In 2014, Bucaneve et al. could show the superiority of piperacillin/tazobactam combined with tigecycline as first-line empiric antibiotic therapy compared to piperacillin/tazobactam alone in patients with hematological malignancies and high-risk neutropenia [17]. Aside from the study of Bucaneve et al. [17] few publications are addressing the question of tigecycline administration as an adjunction to the standard therapies in leukemia patients in high-risk neutropenia. In 2013, Schwab et al. published a study reporting on 35 patients from four different university hospitals who received tigecycline during neutropenic fever under antileukemic treatment [25]. With an overall response rate after 7 days of 43%, defined as resolution of any signs of infection, their response rate is slightly better than our documented overall response rate of 35%. The authors could show that patients in prolonged neutropenia—who are a comparable cohort to our patients—had a lower response rate of only 13% to tigecycline-based regimens, which is significantly worse than in our cohort [25]. In a prospective study conducted by Zhou et al. in 2018, tigecycline was administered to 125 patients with hematological malignancies and neutropenic fever after 72 h of treatment with first-line antibiotics [26]. The authors could observe a high response rate of 68% in this patient cohort [26].

However, in our patient cohort, we observed comparable overall response rates defined by the resolution of fever >38 °C or decrease of CrP >10% in the standard treatment group compared to the tigecycline group, in which vancomycin or linezolid have been changed for tigecycline at a defined time point. The secondary endpoints including infection-associated 30-day mortality, rate of pneumonia, and septic courses also did not differ significantly between the two groups as expected after refutation of the primary endpoint hypothesis. However, higher rates of sepsis and infection-associated mortality were observed in the control group. Compared to the patient population from the Bucaneve et al. study [17], our patient population was neutropenic for a longer period and had received different antimicrobial therapeutic agents before study inclusion. This could be a possible explanation for our results since pathogens—in particular gram-positive pathogenes—were already eradicated by the previous therapies, while the patients from Bucaneve’s cohort were therapy-naïve and received tigecycline in the first-line therapy during the first episode of neutropenic fever. A major difference of our study to the study of Schwab et al. [25] is that in the study of Schwab et al. different antibiotic regimens prior and/or in addition to tigecycline were used and in four patients tigecycline was even administered as a monotherapy. In addition, there was no head-to-head comparison with a standard regimen [25]. Due to the usage of the standard antibiotic regimen in each patient, our patient cohort is more homogeneous, facilitating the interpretation of results. Compared to the high response rate of 68% observed by Zhou et al. [26], our response rate was much lower, but as in the study by Schwab et al. [25], concomitant or previous antibiotic therapy was not standardized in the study by Zhou and colleagues, thereby implying that other antibiotics of last resort could have been applied in addition to tigecycline. Furthermore, there was no control group in this prospective study to compare the overall response and outcome.

Tigecycline is often discussed as salvage therapy for patients with multi-resistant bacteria. Indeed, colonization with multi-resistant bacteria is an increasing problem in leukemia patients [27]. However, in each group of our study, only one patient with a multi-resistant bacterium was detected. In the tigecycline group, a vancomycin-resistant *Enterococcus faecium* (VRE) was detected in the blood culture sample previous to tigecycline administration. Possibly, this could be a subgroup of patients who might profit from tigecycline administration if the bacterium is resistant to linezolid and vancomycin or if linezolid cannot be administered for other reasons. In both groups, all other detected bacteria were not resistant to vancomycin/linezolid and/or tigecycline (including *Staphylococcus epidermidis, Staphylococcus lugdunensis,* and *Citrobacter freundii*) and did not lead to any treatment adjustments. The use of tigecycline in patients with bacteremia is controversially discussed: although tigecycline has a high volume of distribution and penetrates well into different tissues, it has low serum levels, which may result in reduced efficacy in bloodstream infections [22,28,29]. This underlines the need for additional broad-spectrum antibiotics in combination with tigecycline in our patient population besides the above-mentioned gap of efficacy for *Pseudomonas* spp. A positive characteristic of tigecycline is its less nephrotoxicity compared to vancomycin. This could also be a possible indication of the use of tigecycline if linezolid cannot be administered. Nevertheless, creating long-term multiresistant strains by prolonged use of tigecycline in neutropenic patients might be another challenge and should be included in the therapy decision [30].

Whenever comparing our study results with other studies, it is important to keep in mind, that we use ceftazidim instead of piperacillin/tazobactam as first step antibiotic approach during first FUO episode (for treatment schedule, please refer to materials and methods). In general, according to NCCN guideline version 1.2021, piperacillin/tazobactam is highly recommended for treating neutropenic patients in first fever episode, nevertheless, ceftazidim (category 2B according to NCCN guidelines) [31] is more often used at our department due to local piperacillin/tazobactam resistant strains.

A possible observer bias in our study could be a timely regeneration of the bone marrow, as our cohort was already in long-lasting profound neutropenia after multiple escalations of the antimicrobial therapy regimen. We attempted to circumvent this possible bias by including only patients who were still in profound neutropenia at the time point of evaluation. Secondly, there may have been a selection bias since this was a non-randomized study design. The change to tigecycline may have been implemented more frequently in critically ill patients. This may underestimate the effect of tigecycline.

Most likely due to the small patient numbers in our single-center study, we could not observe significant statistical differences in secondary endpoints and further studies should be performed in a larger patient cohort. Nevertheless, we were able to show that tigecycline is at least as effective as standard therapy, showing a benefit regarding sepsis rate and infection-associated mortality in our patient cohort.

## 4. Materials and Methods

### 4.1. Study Population

In this retrospective single-center study, we screened all patients with newly diagnosed, refractory, or relapsed acute leukemia who were under treatment at the department of oncology and hematology at the University Medical Center Hamburg-Eppendorf, Germany, between January 2012 and July 2020. Since treatment of acute leukemia includes several chemotherapeutic cycles, some patients have been enrolled more than once. Patients who underwent conditioning regimens for allogeneic stem cell transplantation or were shortly after transplantation (<100 days) at the time of tigecycline administration have been excluded. Patients have been identified by screening for the ICD 10 diagnostic codes (International Statistical Classification of Diseases and Related Health Problems) C92 (acute myeloid leukemia) and C91 (acute lymphoblastic leukemia) at each admission.

The retrospective data collection was performed in accordance with local legal requirements (§12 Hamburgisches Krankenhausgesetz) and was reviewed and approved by the Ethics Committee of the Medical Council of Hamburg (vote number PV7335). Informed consent was waived by the ethics committee since only pseudonymous data was analyzed and published.

### 4.2. Clinical Data Collection

Data of the clinical courses were extracted from the patient’s electronic medical records (Soarian, Cerner, North Kansas City, MO, USA). Laboratory investigations and clinical imaging including chest X-ray and computed tomography were obtained during clinical routine.

### 4.3. Definition of Time Points and Inclusion Criteria

**Figure 2:**
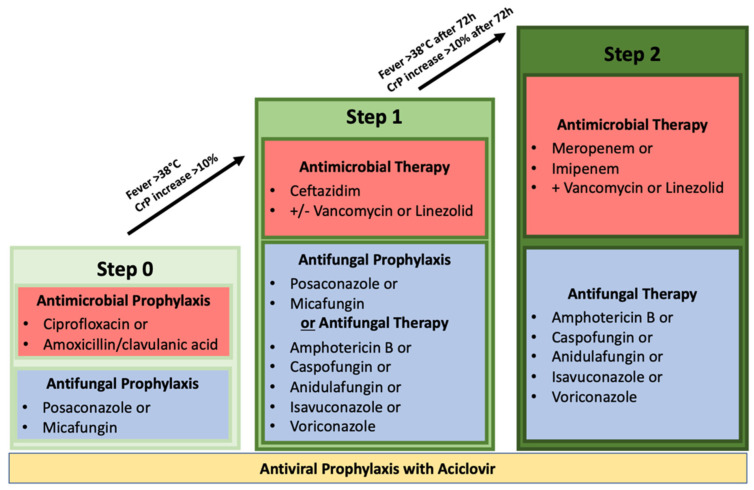
Hospital-based anti-infective escalation schedule. Each patient in this study had to undergo these escalation steps before study inclusion. CrP = C-reactive protein.

All patients had to be at least 18 years old and diagnosed with AML or ALL. Each patient had to be treated according to an anti-infective escalation schedule as usually applied at the department comprising the following agents before being considered for inclusion (refer to Figure 2).

– Step 0: Prophylactic antibiotic administration with ciprofloxacin or amoxicillin/clavulanic acid. Prophylactic antiviral administration with acyclovir. Prophylactic antifungal administration with posaconazole or micafungin;
– Step 1: Neutropenic fever or increase in inflammatory parameters; use of the antibiotic agent ceftazidime with or without vancomycin or linezolid. Switch of antifungal therapeutic therapy was not mandatory but possible. Therapeutic antifungal administrations include amphotericin B, caspofungin, anidulafungin, isavuconazole, and voriconazole. Prophylactic acyclovir administration had to be continued;
– Step 2: Persistent neutropenic fever or increase in inflammatory parameters >72 h after step 1. Switch of antibiotic therapeutics to a carbapenem (either meropenem or imipenem/cilastatin) with concomitant administration of vancomycin or linezolid. Antifungal therapy had to be switched to a therapeutic approach with either amphotericin B, caspofungin, anidulafungin, isavuconazole, or voriconazole. Prophylactic acyclovir administration had to be continued.

Neutropenia was defined as <500 neutrophils/µL according to grade IV neutropenia defined by CTCAE v 5.0 grading [32].

Tigecycline was administered to all patients at 50 mg twice daily intravenously with a loading dosage of 100 mg. In the tigecycline group, the baseline time point was defined as the first administration of tigecycline >72 h after the first carbapenem and vancomycin or linezolid administration with concomitant antifungal therapy and acyclovir prophylaxis (step 2). Tigecycline replaced the treatment with vancomycin or linezolid. Response assessment was performed after 72 h of the first tigecycline administration. In the control group, the baseline time point was defined as 72 h after first the carbapenem and vancomycin or linezolid administration with concomitant antifungal therapy and acyclovir prophylaxis (step 2). Response assessment was performed further 72 h later. At baseline, all patients had to have a fever >38 °C and/or increased c-reactive protein levels (CrP) as a marker for inflammation by >10% after more than 72 h of carbapenem plus vancomycin or linezolid administration and antifungal therapy (step 2) with prophylactic acyclovir administration.

**Figure 3:**
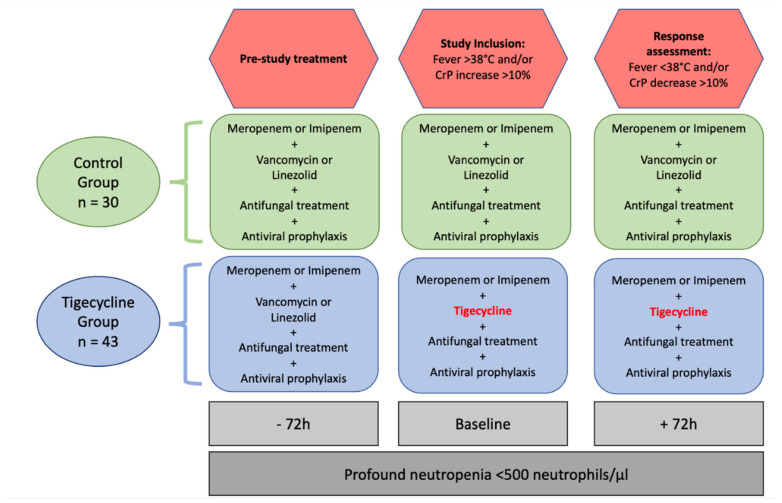
Workflow diagram. All patients have been treated with carbapenem and vancomycin or linezolid plus antifungal therapy plus antiviral prophylaxis for 72 h. At baseline, all patients had fever >38 °C and/or CrP increase of >10%. In the control group, treatment proceeded and in the tigecycline group, vancomycin or linezolid was switched to tigecycline. After another 72 h hours, response to treatment was assessed, defined as resolution of fever (<38 °C) or decrease of CrP level >10% compared to baseline. CrP = C-reactive protein.

All patients must have profound neutropenia at baseline and at the timepoint of response assessment to exclude regeneration of leukocyte count as a confounder for response assessment. Other antibiotics of last resort were prohibited (for visualization of workflow refer to Figure 3). According to the hospital-based standard procedure of performing low-dose computed tomography of the chest and collection of blood and urinary cultures in every patient with multidrug-resistant neutropenic fever, each patient of our cohort underwent these diagnostic steps.

### 4.4. Primary and Secondary Endpoints

The primary endpoint of this retrospective study is the overall response rate defined by the resolution of fever (defined as <38 °C) and concomitant CrP decrease by more than 10% compared to baseline 72 h after the first dose application of tigecycline for the tigecycline group and 72 h after the first day of occurrence of multidrug-resistant fever for the control group, respectively.

Secondary endpoints were the number of septic courses, the incidence of pneumonia, and 30-day mortality from baseline.

### 4.5. Statistical Analysis

All statistical analyses were performed by using the Statistical Package for Social Sciences statistical software, version 21.0 (IBM Corp., Armonk, New York, NY, USA) and GraphPad Prism version 9.2.0, USA. Continuous values are presented as median with interquartile range (IQR). Categorical variables are expressed as numbers (%) and compared by Fisher’s exact test. A *p*-value < 0.05 was considered statistically significant. The reported *p*-values are two-tailed.

## 5. Conclusions

In conclusion, switching the antibiotic approach from vancomycin or linezolid to tigecycline in combination therapy with a carbapenem and antifungal treatment at persistent multidrug-resistant neutropenic fever showed a benefit in this retrospective study regarding sepsis rate (47% vs. 33%) and infection-associated 30-day mortality (13% vs. 5%) in our patient cohort, although being non-significant due to the small cohort size but showing a clear trend towards tigecycline therapy. No differences were observed regarding resolution of fever and decrease of CrP-level (*p* = 0.476), indicating that tigecycline is not inferior to the previous standard treatment with vancomycin or linezolid in combination with a carbapenem at this point and seems to be a good alternative, especially for those patients who cannot receive vancomycin or linezolid by any reasons.

## Figures and Tables

**Table 1:** Demographic information, underlying hematological malignancy, remission status, and disease-specific therapy of patients in the control and in the tigecycline group.

	Control Group (*n* = 30)	Tigecycline Group (*n* = 43)
Male, no. (%)	16 (53)	25 (58)
Age, median (IQR)	60.5 (44–68)	59 (43–68)
Relevant comorbidities, no. (%)	8 (27)	12 (28)
Cerebrovascular disease	/	1 (2)
Breast cancer	3 (10)	4 (9)
Bladder cancer	/	1 (2)
Prostate cancer	/	2 (5)
Lung emphysema	/	1 (2)
Multiple Myeloma	/	1 (2)
Acute myeloid leukemia	/	1 (2)
Diabetes mellitus II	2 (7)	1 (2)
Peripheral artery disease	1 (3)	/
Coronary heart disease	2 (7)	/
Charlson-Comorbidity-Index (CCI), median (IQR) ^1^	4 (2–5)	4 (2–5)
Underlying hematological malignancy, no. (%)		
Acute myeloid leukemia	25 (83)	37 (86)
Acute lymphoblastic leukemia	5 (17)	6 (14)
Remission status, no. (%)		
First diagnosis	25 (83)	33 (77)
Relapse/refractory	5 (17)	10 (23)
Under intensive treatment, no. (%)	28 (93)	38 (88)
Duration (days) of profound neutropenia (<500 neutrophils/µL), median (IQR)	25 (20–28)	21 (17–30)

^1^ = according to Schneeweiss et al. [23]; IQR = interquartile range.

**Table 2:** Overview of the distribution of antibiotic therapies in the two groups.

	Control Group (*n* = 30)	Tigecycline Group (*n* = 43)
Carbapenem, no. (%)	30 (100)	43 (100)
Meropenem	27 (90)	43 (100)
Imipenem/Cilastatin	3 (10)	0 (0)
Vancomycin, no. (%)	26 (87)	41 (95)
Linezolid, no. (%)	13 (43)	13 (30)
Vancomycin and linezolid sequentially, no. (%)	10 (33)	11 (26)

**Table 3:** Characteristics of disease courses and detected microbes in patients treated with or without tigecycline.

	Control Group (*n* = 30)	Tigecycline Group (*n* = 43)	*p*-Value
Overall response rate, no. (%)	13 (43)	15 (35)	0.476
Infection-associated 30-day mortality, no. (%)	4 (13)	2 (5)	not done
Sepsis, no. (%)	14 (47)	14 (33)	not done
Pneumonia in X-ray or computed chest tomography, no. (%)	16 (53)	26 (60)	not done
Catheter-associated infections, no. (%)	4 (13)	16 (37)	not done
Infections of urinary tract, no. (%)	3 (10)	6 (14)	not done
Positive blood culture samples, no. (%)	9 (30)	22 (51)	not done
*Staphylococcus haemolyticus*	4 (13) *	5 (12)	
*Staphylococcus hominis*	1 (3)	4 (9)	
*Staphylococcus epidermidis*	4 (13)	9 (21)	
*Staphylococcus lugdunensis*	/	1 (2)	
*Stenotrophomonas maltophilia*	/	2 (5)	
*Citrobacter freundii*	/	1 (2)	
*Enterococcus faecium* (non-VRE)	/	3 (7)	
*Enterococcus faecalis* (non-VRE)	1 (3)	1 (2)	
*Enterococcus faecium* (VRE)	/	1 (2)	
*Escherichia coli*	/	1 (2)	
*Pseudomonas aeruginosa*	1 (3)	/	
Positive BAL, no. (%)	15 of 18 (83)	8 of 13 (66)	
Yeasts	3 (10)	7 (16)	
Coagulase-neg. *staphylococci*	5 (17)	10 (23)	
*Enterococcus* spp.	4 (14)	3 (7)	
Herpes simplex virus 1	2 (7)	2 (5)	
Enterovirus	1 (3)	/	
Rhinovirus	1 (3)	/	
*Streptococcus viridans*	/	4 (9)	
*Metapneumonia virus*	/	1 (2)	
*Aspergillus fumigatus*	/	1 (2)	
*Escherichia coli*	/	1 (2)	

* in one sample, a tigecycline-resistant *Staphylococcus haemolyticus* was detected. *p*-values are 2-sided, calculated with Fisher’s exact test. BAL = bronchoalveolar lavage; VRE = Vancomycin-resistant *Enterococcus*.

## Data Availability

The datasets generated during and/or analyzed during the current study are available from the corresponding author on reasonable request.
